# Supplementary material for: Determination of Tear Lipid Film Thickness Based on a Reflected Placido Disk Tear Film Analyzer
Source: Diagnostics (Basel). 2020 May 28;10(6):353. doi: 10.3390/diagnostics10060353 (PMC7345488; doi:10.3390/diagnostics10060353)

**Supplementary file 2**

**The standard procedures to decompose dynamic lipid film recording for obtaining image sequences for each subject**

Using the function of capture consecutive image in a video player “PotPlayer” to produce the image frame with the time interval 0.05 seconds

1. Open the PotPlayer

**
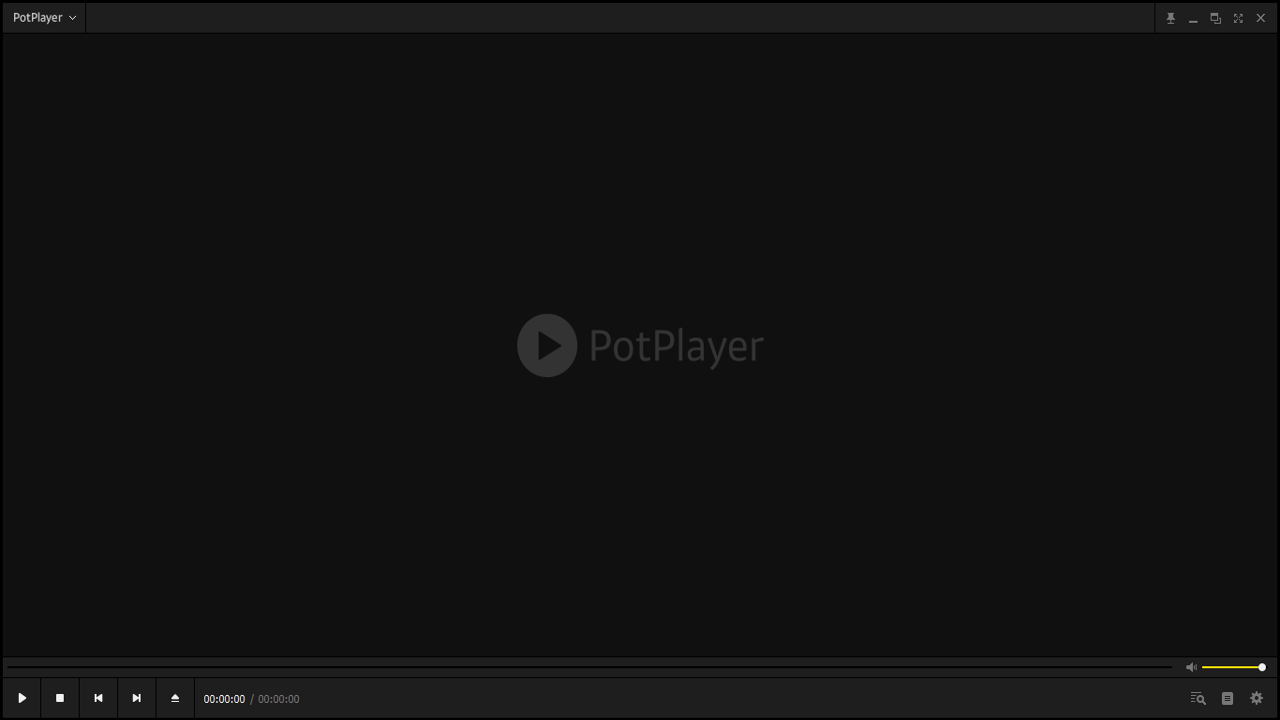
**

1. Open the video recording file assessed by Keratograph® 5M

**
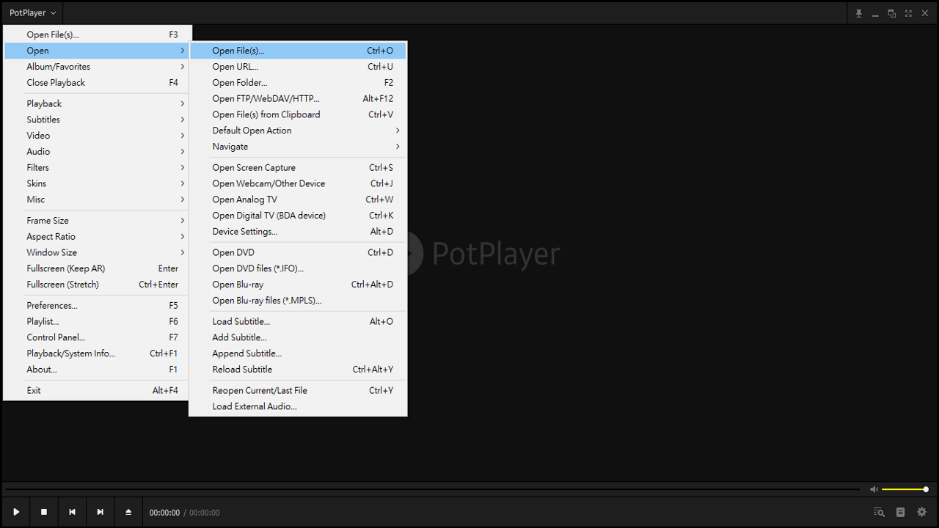
**

1. Capture consecutive images

**
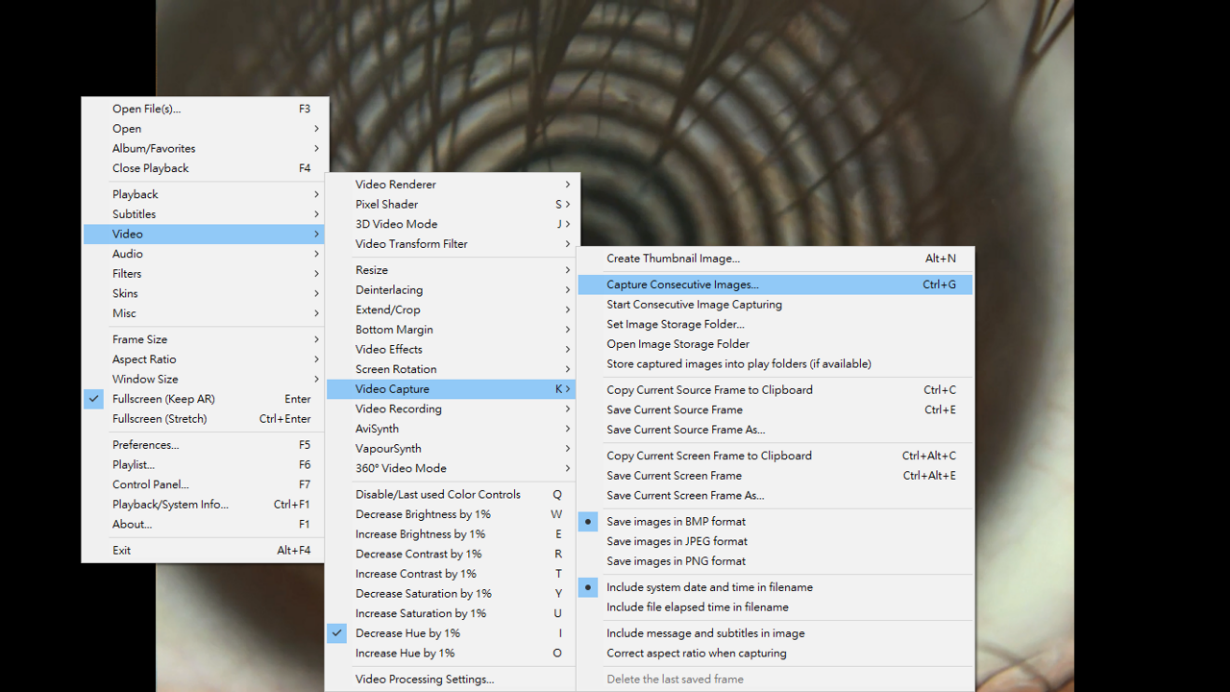
**

1. Select every 50 ms and start


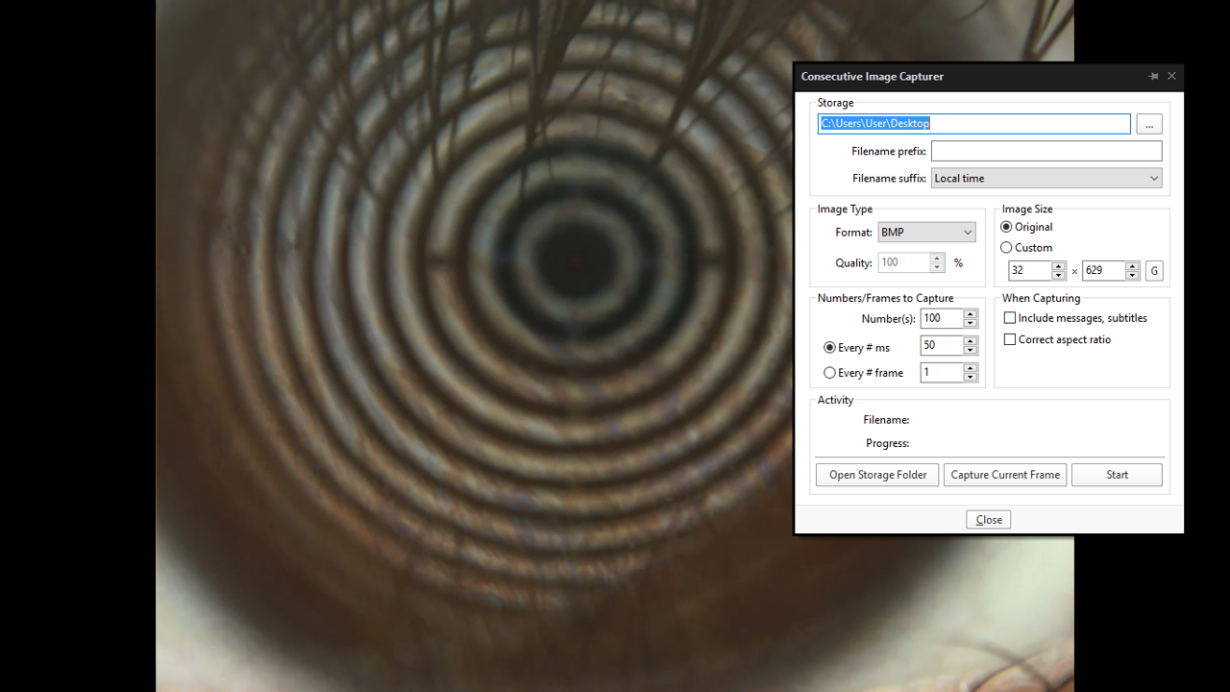

Supplement: Supplementary file 1 [file diagnostics-10-00353-s001.zip › Supplementary file 2.docx]
