# Supplementary material for: Determination of Tear Lipid Film Thickness Based on a Reflected Placido Disk Tear Film Analyzer
Source: Diagnostics (Basel). 2020 May 28;10(6):353. doi: 10.3390/diagnostics10060353 (PMC7345488; doi:10.3390/diagnostics10060353)
Supplement: Supplementary file 1 [file diagnostics-10-00353-s001.zip › Supplementary file 3.docx]

**Supplementary file 3**

**Using ImageJ to obtain region of interests (ROIs) of non-segmented and post-segmented color interference lipid film images**

1. Using ImageJ to obtain the ROI image of non-segmented lipid film image by following steps:

| 1. Erase the region within the 2^nd^ bright light circle by Oval selection tool and Clear function in the Edit menu | 1. Erase the region outside the 9^th^ bright light circle by Oval selection tool and Crop function in the Image menu |
| --- | --- |
| 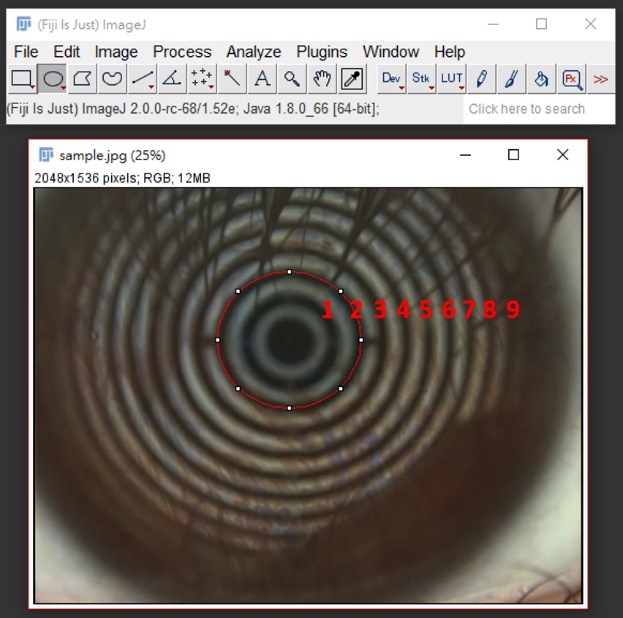 | 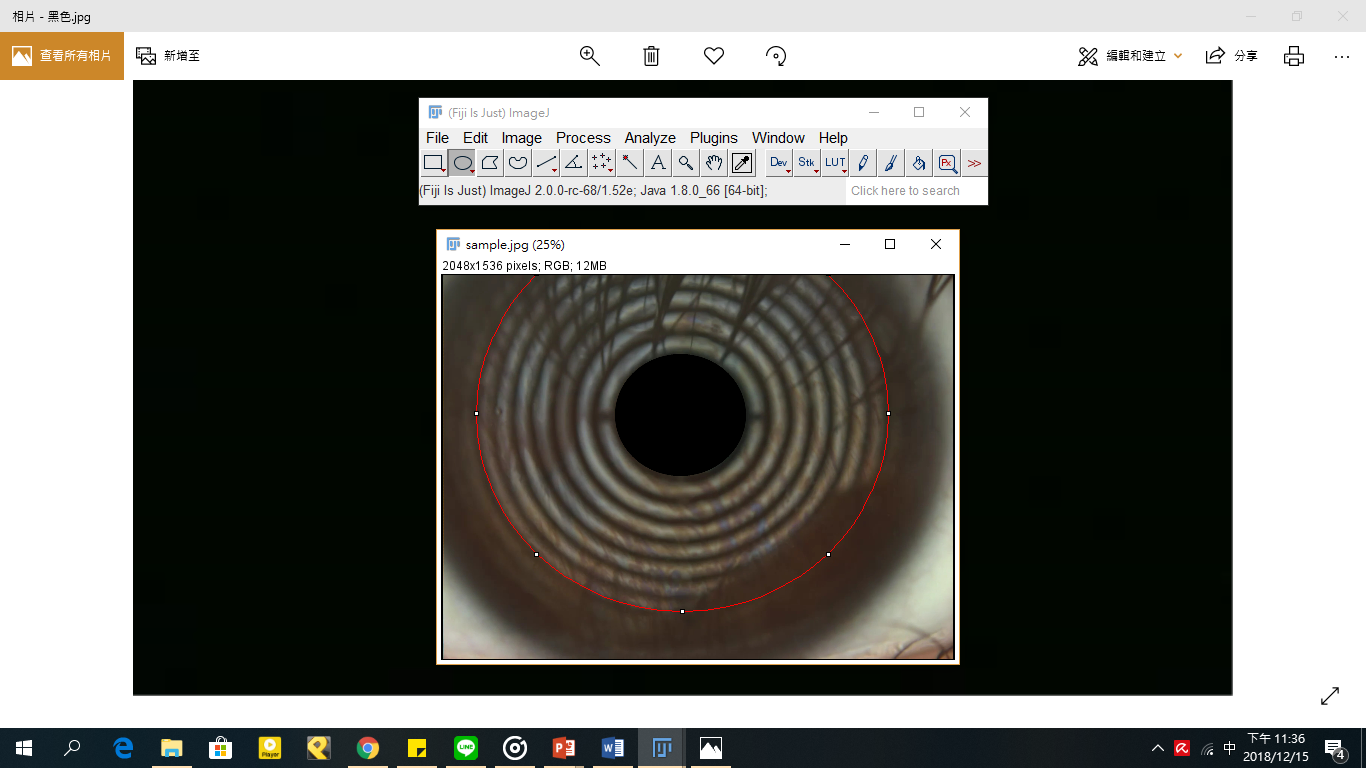 |
| 1. Keep the inferior half circular region by Rectangle selection tool and Crop function in the Image menu | 1. The ROI of the non- or pre-segmented lipid film image is now obtained |
| 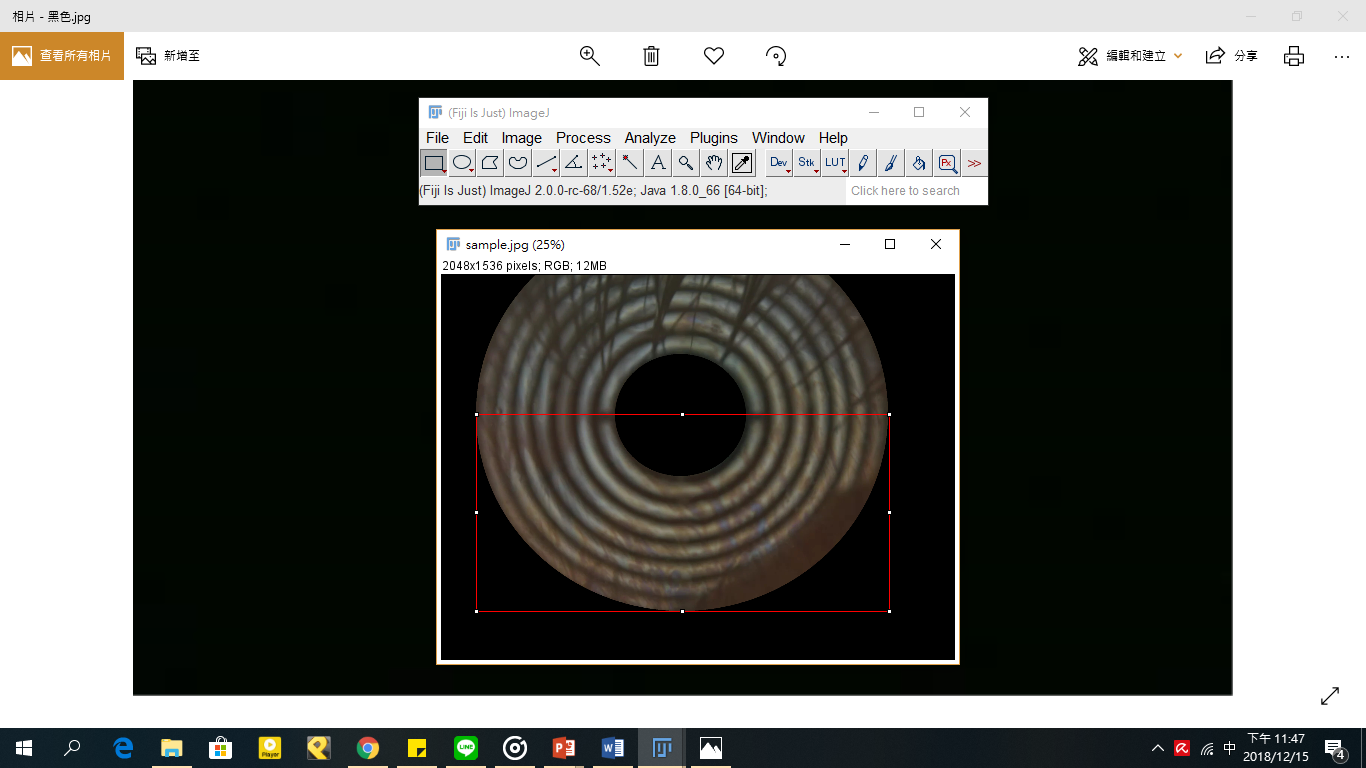 | 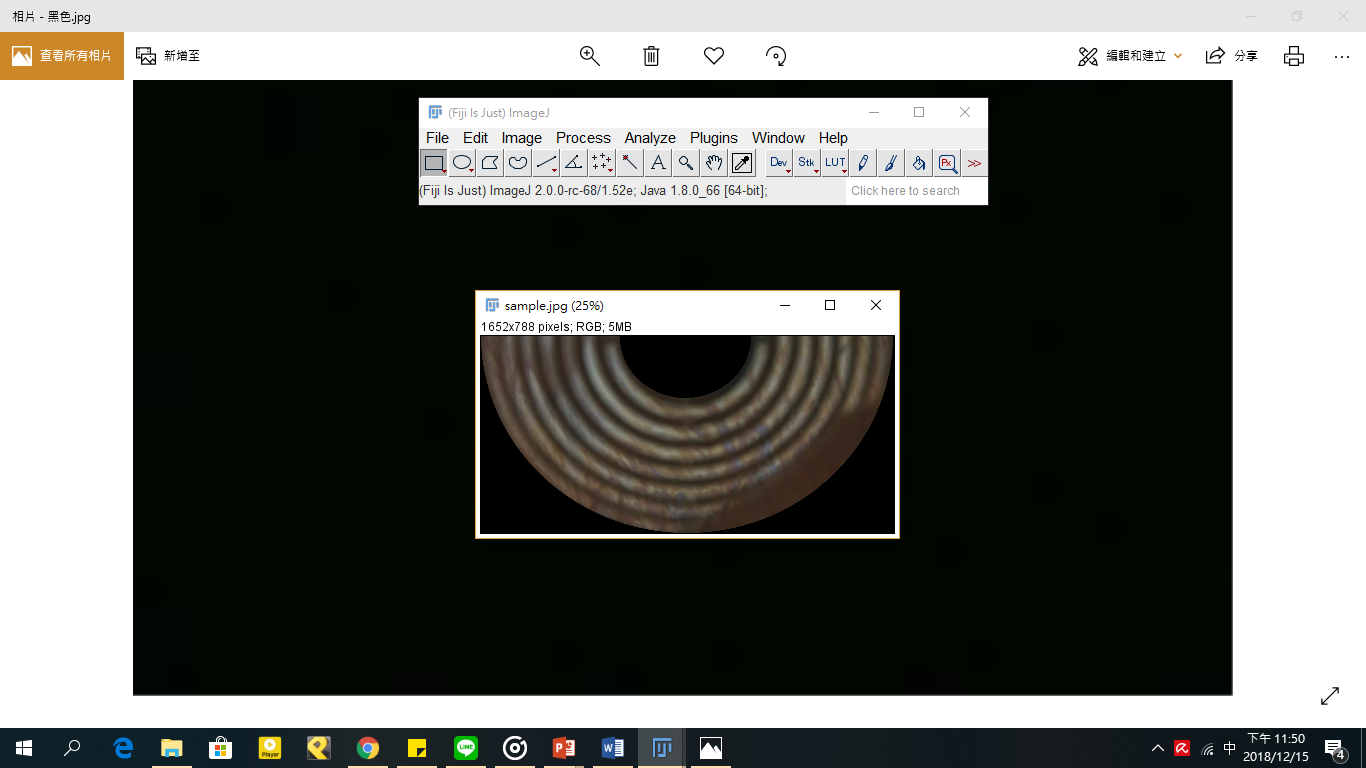 |

1. Using the ImageJ to obtain the ROI of non-segmented lipid film image by following steps:

| 1. Open the ROI image of non-segmented lipid film image | 1. Enter the color threshold options by clicking Image menu 🡪 Adjust 🡪 Color Threshold… |
| --- | --- |
| 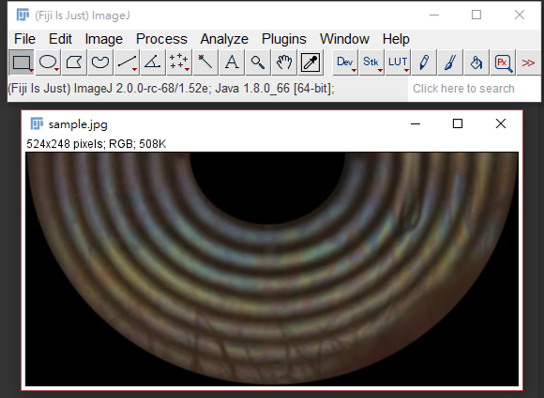 | 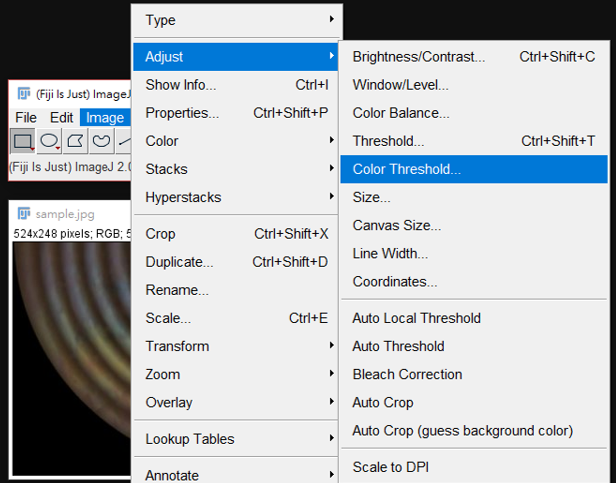 |
| 1. Select MaxEntropy as the Thresholding method, HSB as color space, and check Dark background | 1. Click the Select function in the inferior panel |
| 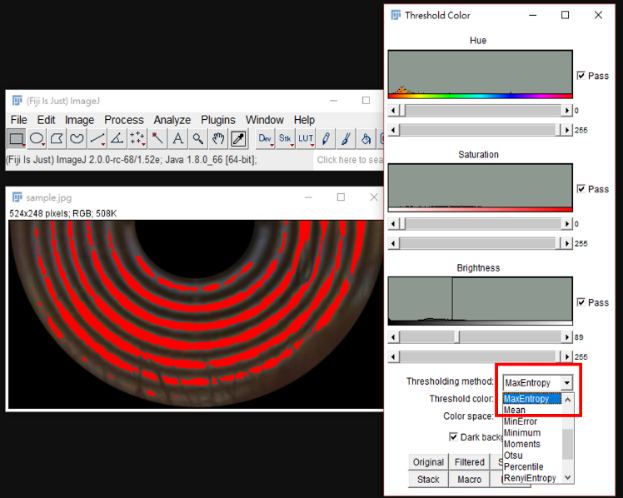 | 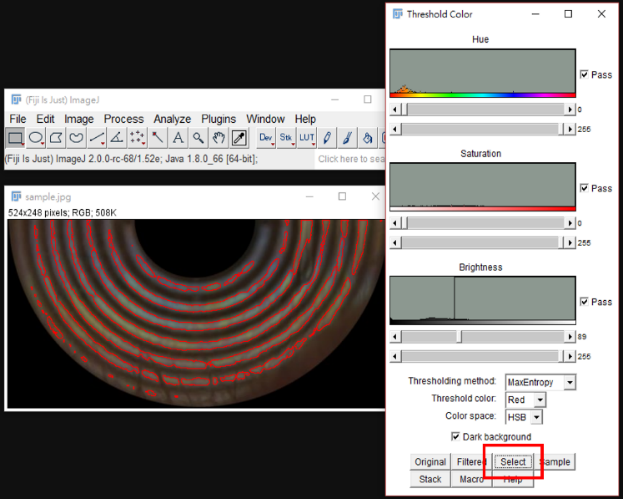 |
| 1. Erase the non-selected region by Clear Outside function in the Edit menu | 1. The ROI of the segmented lipid film image is now obtained |
| 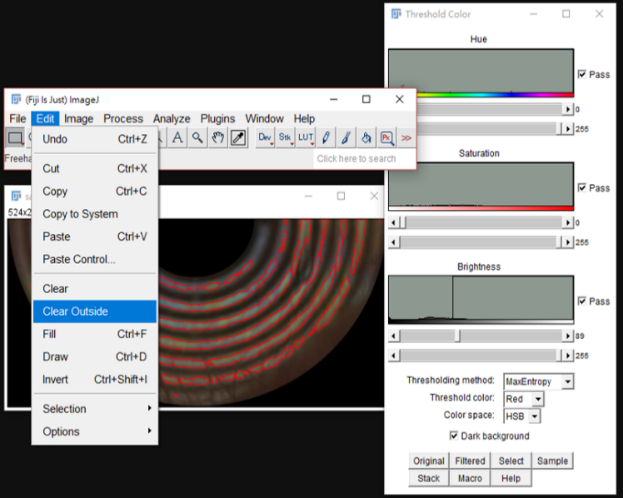 | 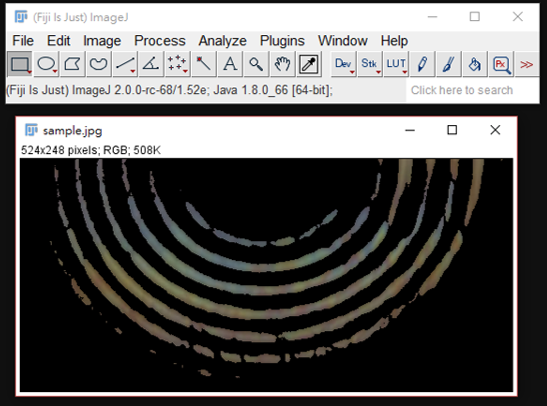 |
