# Supplementary material for: Determination of Tear Lipid Film Thickness Based on a Reflected Placido Disk Tear Film Analyzer
Source: Diagnostics (Basel). 2020 May 28;10(6):353. doi: 10.3390/diagnostics10060353 (PMC7345488; doi:10.3390/diagnostics10060353)

**Supplementary file 5**

**The procedure to determine the lipid layer thickness (LLT) at each pixel coordinate and obtain the average LLT within the region of interest (ROI) of a lipid film image**

1. Open the ROI image of a subject for determining LLT


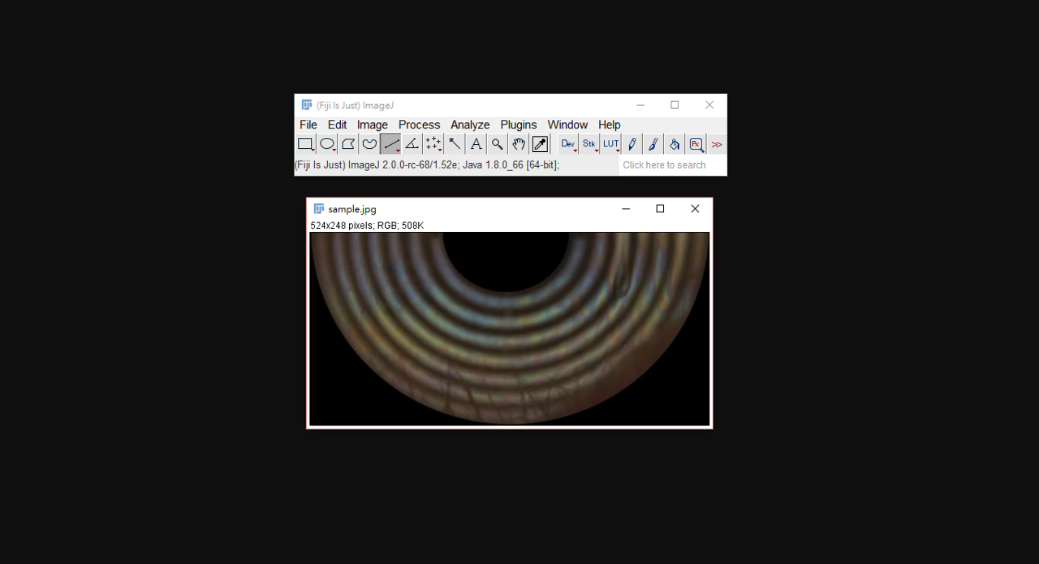


1. Separate the ROI image into 8-bit R, G, B images by Image menu 🡪 Color 🡪 Slit Channels


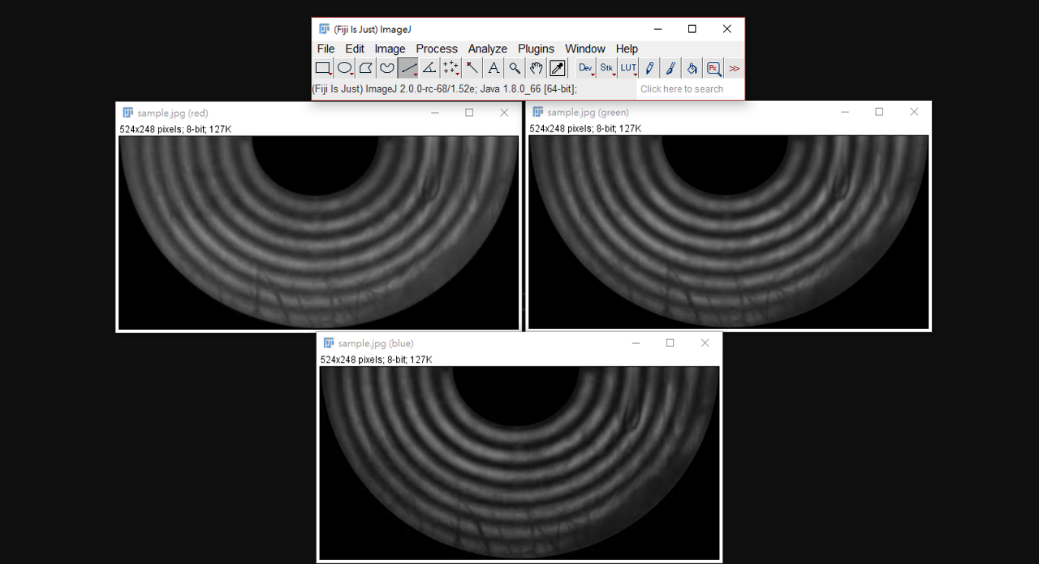


1. Save each image as text image by File menu 🡪 Save as 🡪 Text Image


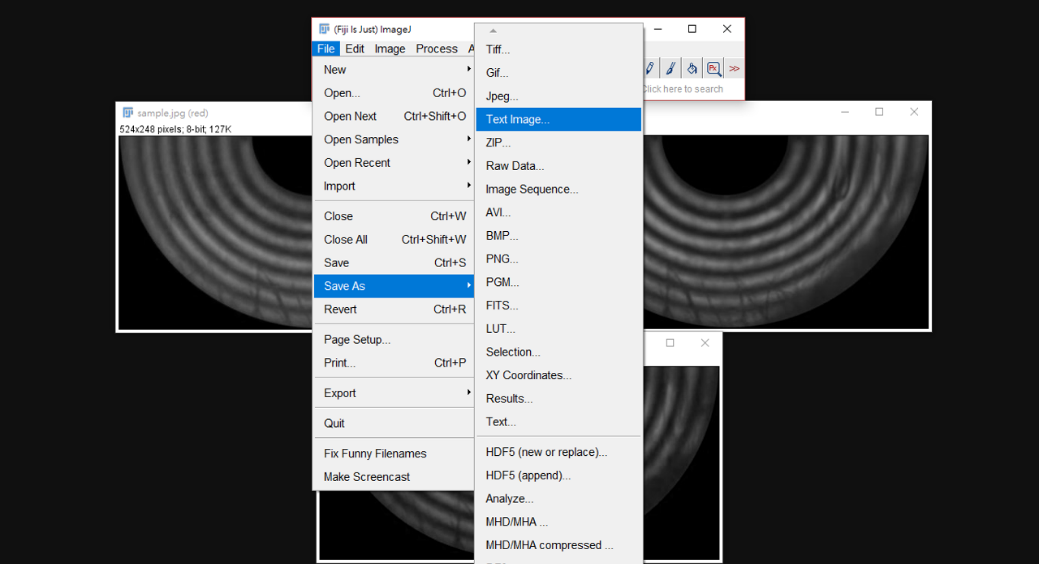


1. Load the text image of R channel into the worksheet **sampleR** in the Excel look-up table (supplementary file 4)


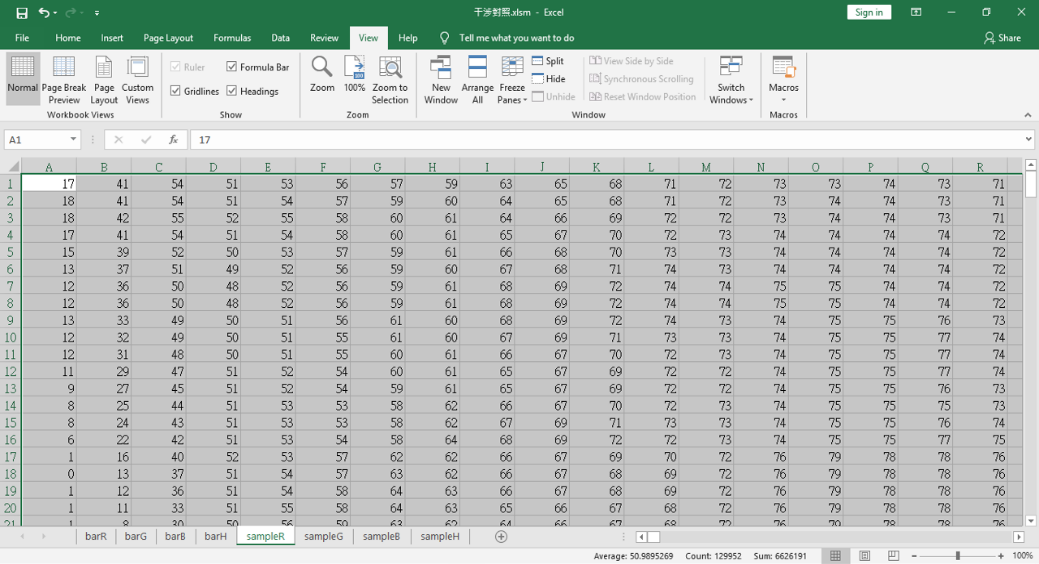


1. Load the text image of G channel into the worksheet **sampleG** in the same Excel look-up table


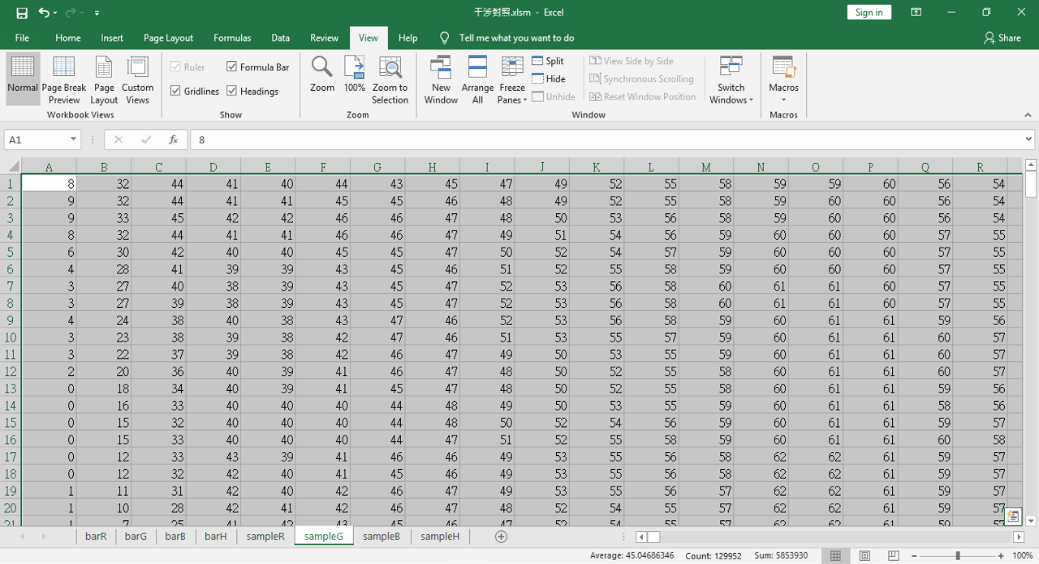


1. Load the text image of B channel into the worksheet **sampleB** in the same Excel look-up table


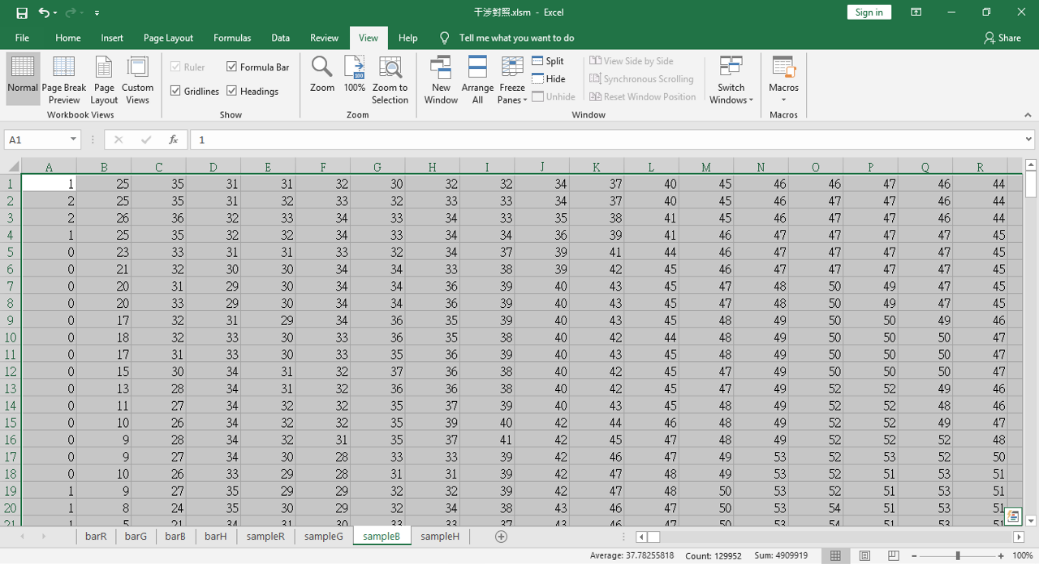


1. Run the Macros **LLT** in the worksheet **sampleH** to determine LLT at each pixel coordinate


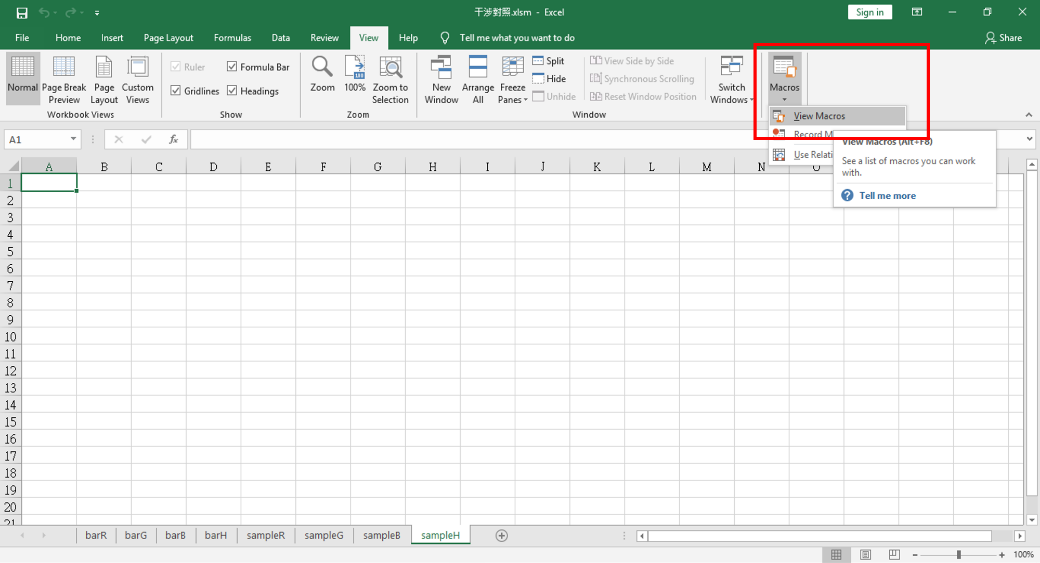


1. LLT of each pixel coordinate is now obtained


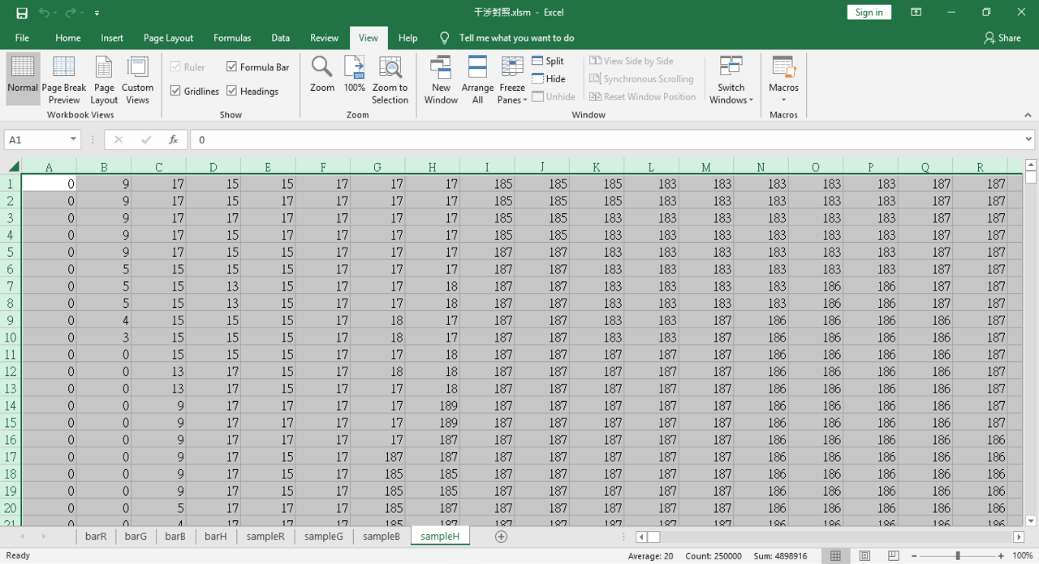


1. Compute the average LLT


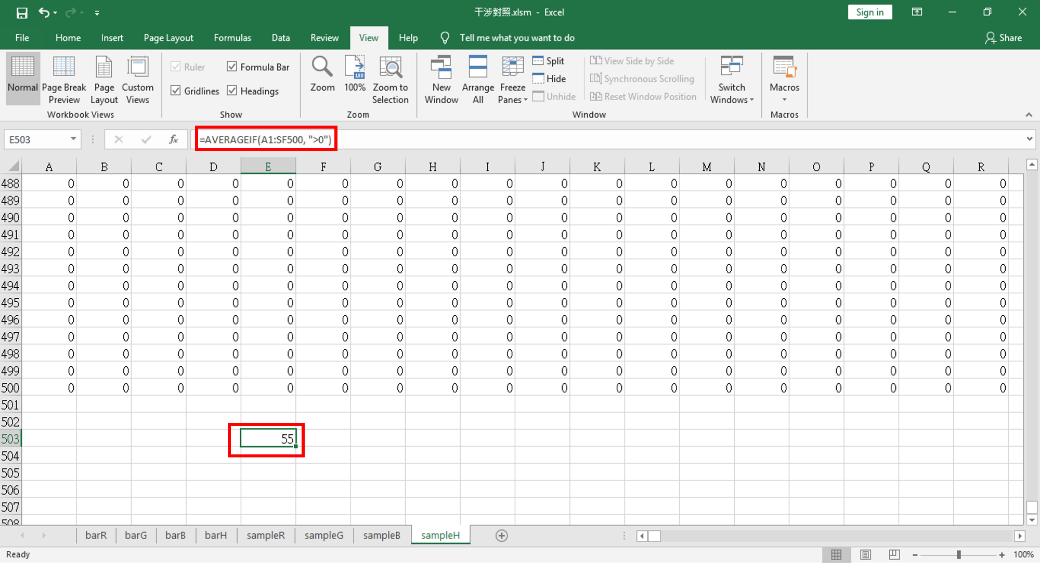

Supplement: Supplementary file 1 [file diagnostics-10-00353-s001.zip › Supplementary file 5.docx]
