# Supplementary material for: Determination of Tear Lipid Film Thickness Based on a Reflected Placido Disk Tear Film Analyzer
Source: Diagnostics (Basel). 2020 May 28;10(6):353. doi: 10.3390/diagnostics10060353 (PMC7345488; doi:10.3390/diagnostics10060353)
Supplement: Supplementary file 1 [file diagnostics-10-00353-s001.zip › Supplementary file 7.docx]

**Supplementary file 7**

**The instruction of the application software in the supplementary file 6: for obtaining the average lipid layer thickness (LLT) of a lipid film image after selection of region of interest (ROI)**

1. Open the supplementary file 6


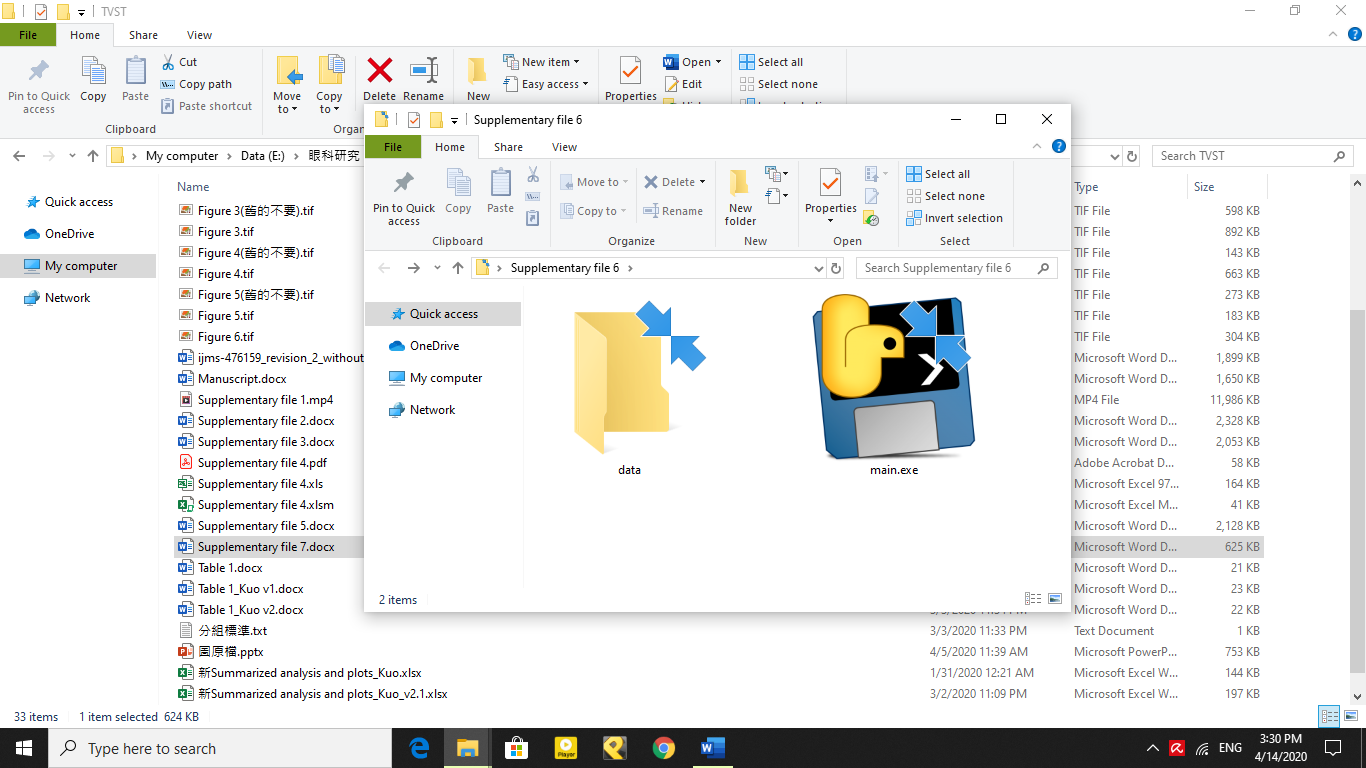


1. Drag the lipid film images after ROI selection into the “data” folder (The name of the file should be named by English or number)


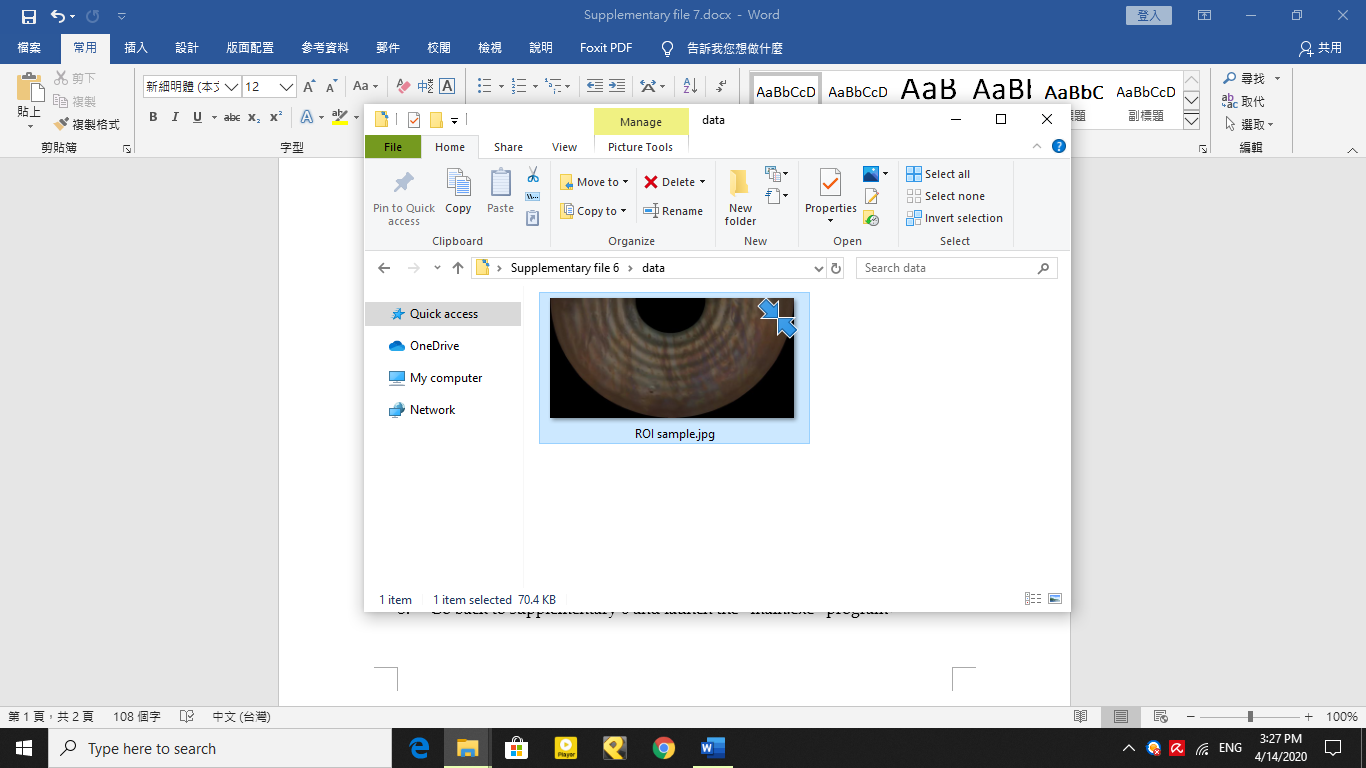


1. Go back to supplementary 6 and launch the “main.exe” program


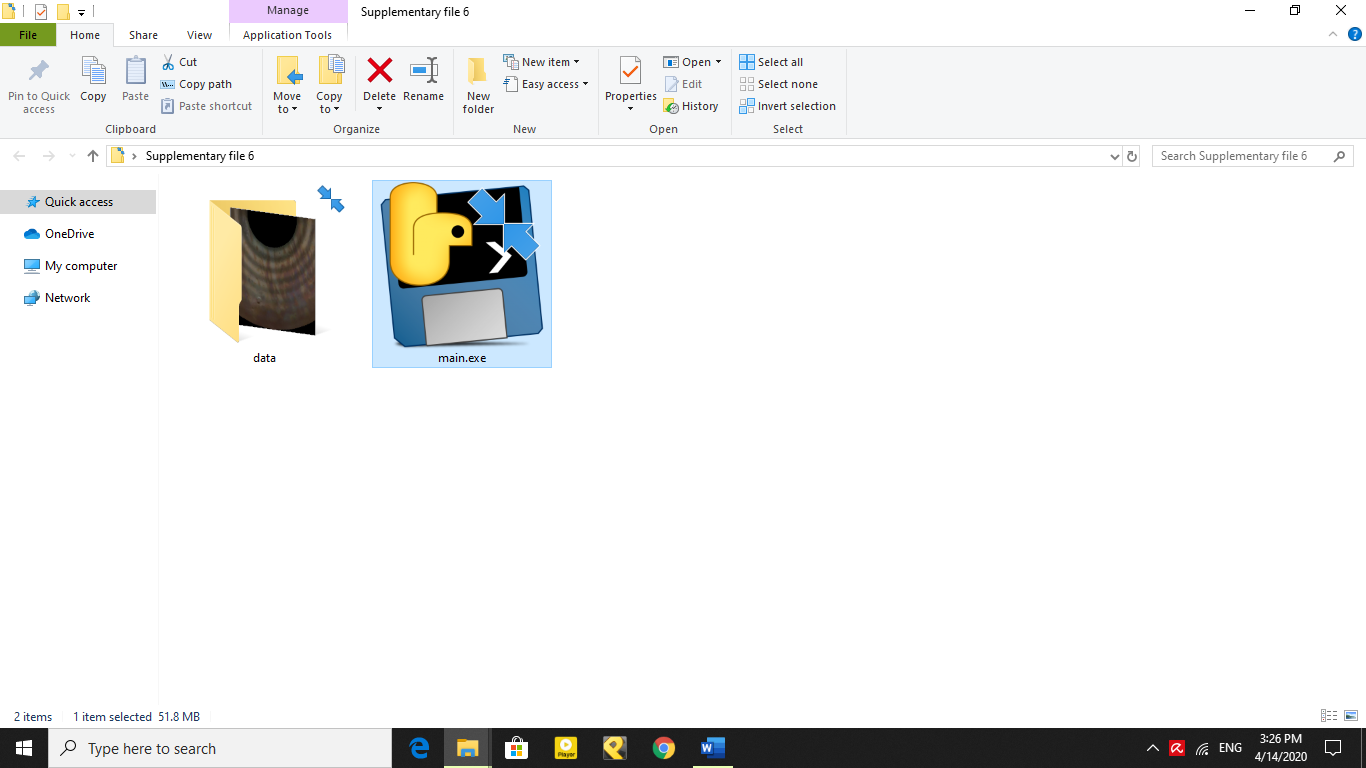


1. After the program calculate the data within 10 s, the program window will close spontaneously. The value of the LLT is then shown in the file named as lipid layer thickness.txt.


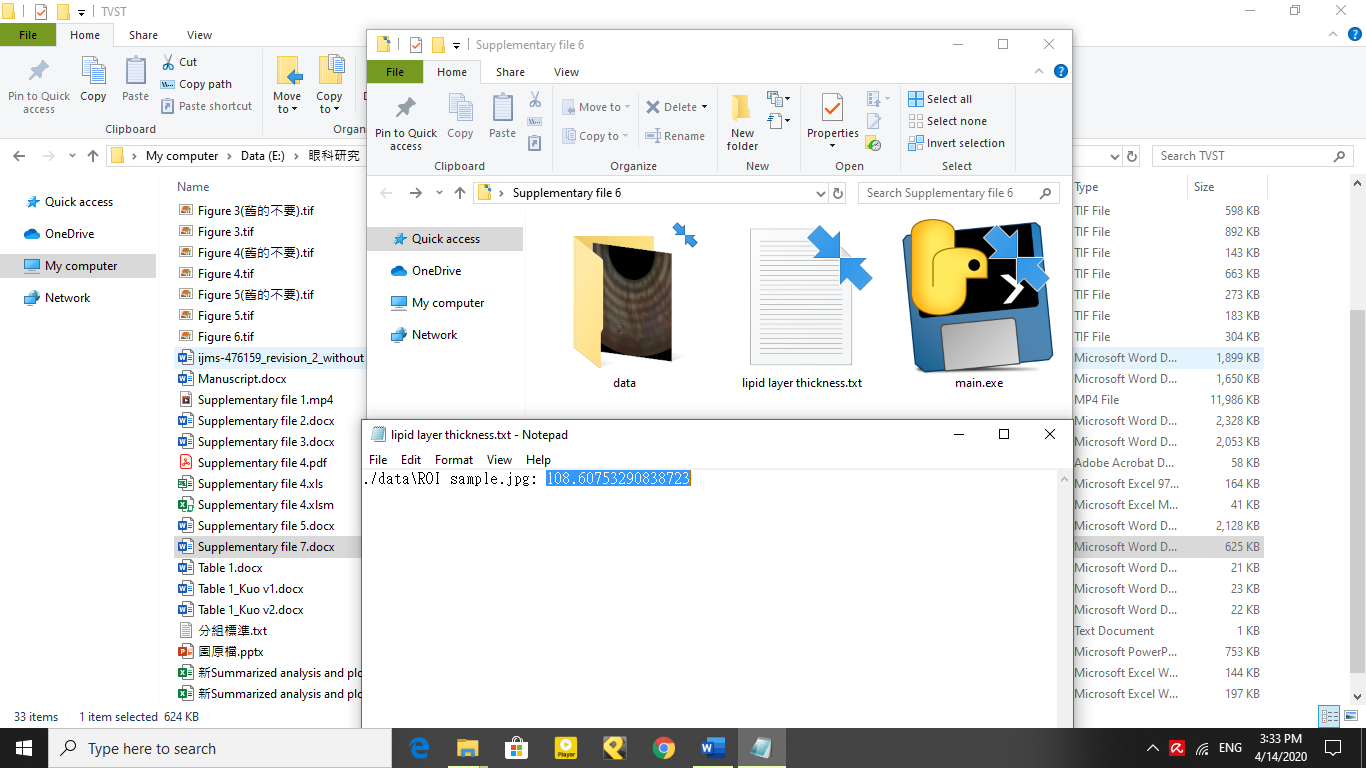


the estimated tear lipid layer thickness is about 108.6 nm
