# Supplementary figures and images for: Determination of Tear Lipid Film Thickness Based on a Reflected Placido Disk Tear Film Analyzer
Source: Diagnostics (Basel). 2020 May 28;10(6):353. doi: 10.3390/diagnostics10060353 (PMC7345488; doi:10.3390/diagnostics10060353)

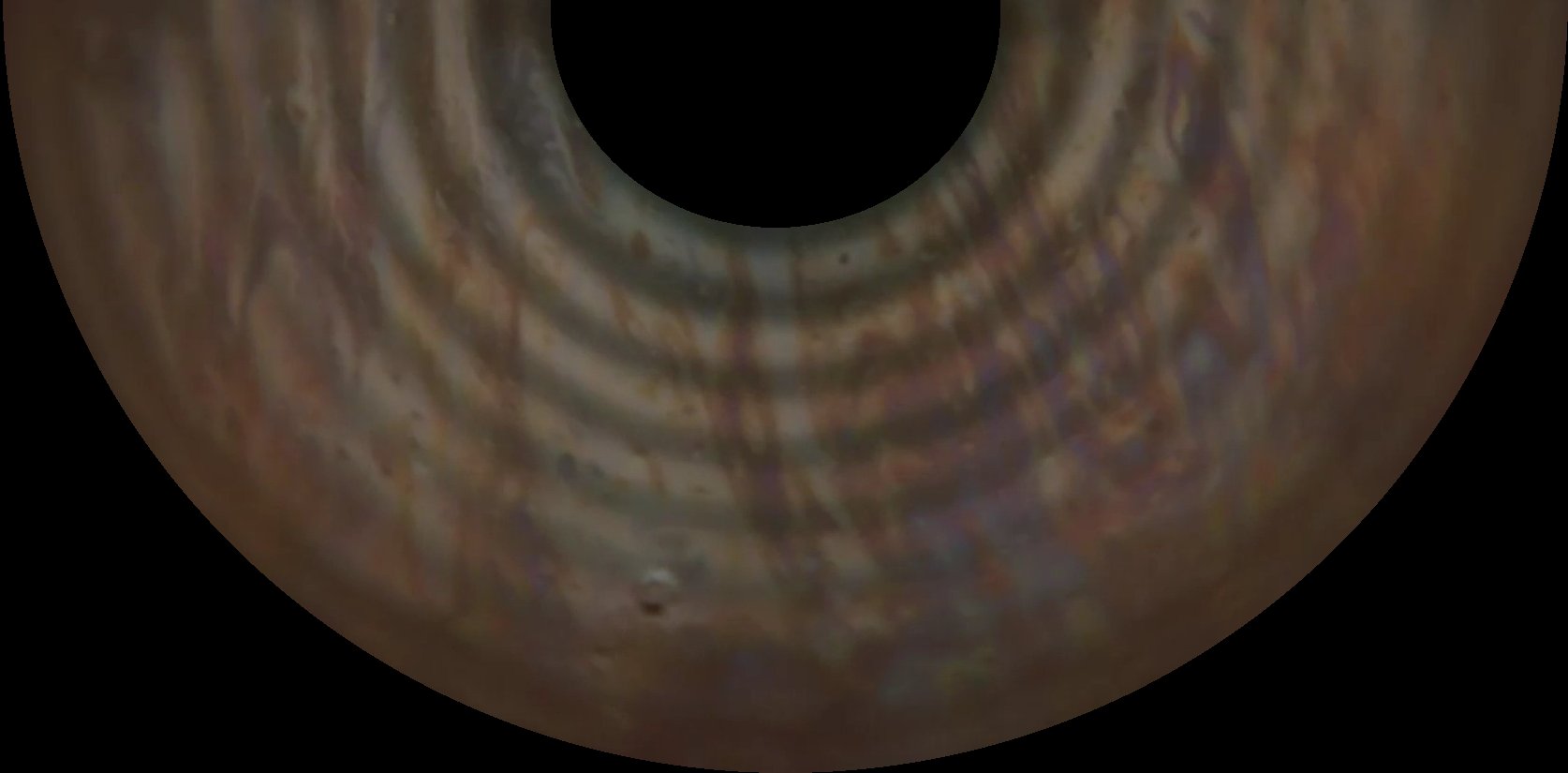

Supplement: Supplementary file 1 [file diagnostics-10-00353-s001.zip › Supplementary file 6/data/ROI sample.jpg]
